# Deep learning assisted single particle tracking for automated correlation between diffusion and function

**DOI:** 10.21203/rs.3.rs-3716053/v1

**Published:** 2024-02-02

**Authors:** Nikos Hatzakis, Jacob Kaestel-Hansen, Marilina de Sautu, Anand Saminathan, Gustavo Scanavachi, Ricardo Correia, Annette Juma Nielsen, Sara Bleshoey, Wouter Boomsma, Tomas Kirchhausen

## Abstract

Sub-cellular diffusion in living systems reflects cellular processes and interactions. Recent advances in optical microscopy allow the tracking of this nanoscale diffusion of individual objects with an unprecedented level of precision. However, the agnostic and automated extraction of functional information from the diffusion of molecules and organelles within the sub-cellular environment, is labor-intensive and poses a significant challenge. Here we introduce DeepSPT, a deep learning framework to interpret the diffusional 2D or 3D temporal behavior of objects in a rapid and efficient manner, agnostically. Demonstrating its versatility, we have applied DeepSPT to automated mapping of the early events of viral infections, identifying distinct types of endosomal organelles, and clathrin-coated pits and vesicles with up to 95% accuracy and within seconds instead of weeks. The fact that DeepSPT effectively extracts biological information from diffusion alone illustrates that besides structure, motion encodes function at the molecular and subcellular level.

### INTRODUCTION

Direct observation of cellular processes is now routinely achieved by fluorescence microscopy and single-particle tracking (SPT) techniques[1–8]. These techniques offer the necessary spatiotemporal resolution to localize and track diffusion of individual biomolecules—from small proteins and viruses to organelles or entire cells—in both 2D and 3D environments[4,5,9–12]. The observed diffusion is highly complex and exhibits considerable spatiotemporal and interparticle heterogeneity, reflecting various biological factors such as internalization stages, local environment, oligomerization states, and interactions with elements like the cytoskeleton, membranes, molecular motors, organelles and more[4,10,13,14]. This intrinsic heterogeneity presents a significant analytical challenge and remains a major bottleneck for extracting quantitative insights from single-particle studies (SPT).

Traditionally, extracting diffusional behavior from SPT experiments relies on fitting the mean squared displacement (MSD) [14–17], or more recently using machine learning[18–21]. These approaches often transform entire trajectories into a single descriptor like the diffusion coefficient, thus averaging out vital temporal information that is essential to interpret biological processes. Achieving temporal segmentation—a prerequisite for unlocking the rich temporal data inherent in biological processes—is a considerable challenge. For instance, manual annotation requires significant expertise and is prohibitively time-consuming for large datasets, and to date is done primarily in 2D. Methods like Rolling MSD[14–17,22] and divide-and-conquer[23] offer automated temporal segmentation, but they are reliant on windowing tracks which introduces a trade-off in temporal sensitivity and accuracy, and they also depend on user-defined, system-specific parameters. Hidden Markov Models[24–27] can segment traces but only if a diffusional metric, often step length, varies significantly between states[27,28].

Current state-of-the-art tools based on machine learning[28–34] can distinguish between diffusional states in 2D with distinct a priori, user-defined diffusional characteristics. While these tools operate well for their specific set of states, they remain less explored for systems displaying multiple, broadly distributed diffusional characteristics common in complex cellular environments and often do not extend to 3D. Achieving temporal segmentation of such heterogeneous behavior is crucial for overcoming the current analytical bottleneck in SPT, yet alone is not enough to decode correlations between heterogeneous behavior, biological context, function, and biomolecular identity.

Identifying biomolecular identity, colocalizing partners, cellular localization, or the time point of a biological event is a challenge in fluorescence microscopy, necessitating specialized analysis, parallelized multicolor and often super-resolution imaging[35–38]. Such experimental design, specialized analysis and fluorescent tagging of biomolecular entities is substantially labor- and material intensive and risks impairing biological function by tagging[39]. These challenges are further compounded by the limitation of using 2-3 imaging channels for quantitative imaging due to spectral overlap. Temporal analysis of diffusional behavior alone could overcome these challenges by acting as an orthogonal probe for extracting biological function, colocalization or identity, minimizing the need for fluorescent tagging, thereby simplifying experimental workflows. However, to date dissecting heterogeneous diffusional behavior remains largely untapped

Here we introduce DeepSPT, a versatile deep learning-based toolbox designed for the rapid, accurate, and automatic temporal analysis of behavior in single-particle tracking (SPT). DeepSPT facilitates extraction of biological insights from 2D or 3D traces solely based on the diffusional characteristics of the tracked objects. The pre-trained DeepSPT pipeline is available as open-source code on GitHub.

[1]Department of Chemistry University of Copenhagen, [2]Center for 4D cellular dynamics, Department of Chemistry University of Copenhagen, [3]Novo Nordisk Center for Optimised Oligo Escape [4]Novo Nordisk foundation Center for Protein Research, [5]Biological Chemistry and Molecular Pharmaceutics Harvard Medical School, [6]Laboratory of Molecular Medicine Boston Children's Hospital, [7]Department of Cell Biology Harvard Medical School, [8]Department of Pediatrics Harvard Medical School, [9]Program in Cellular and Molecular Medicine Boston Children's Hospital, [10]Department of Computer Science University of Copenhagen. For correspondence: e-mail kirchhau@crystal.harvard.edu, e-mail: hatzakis@chem.ku.dk

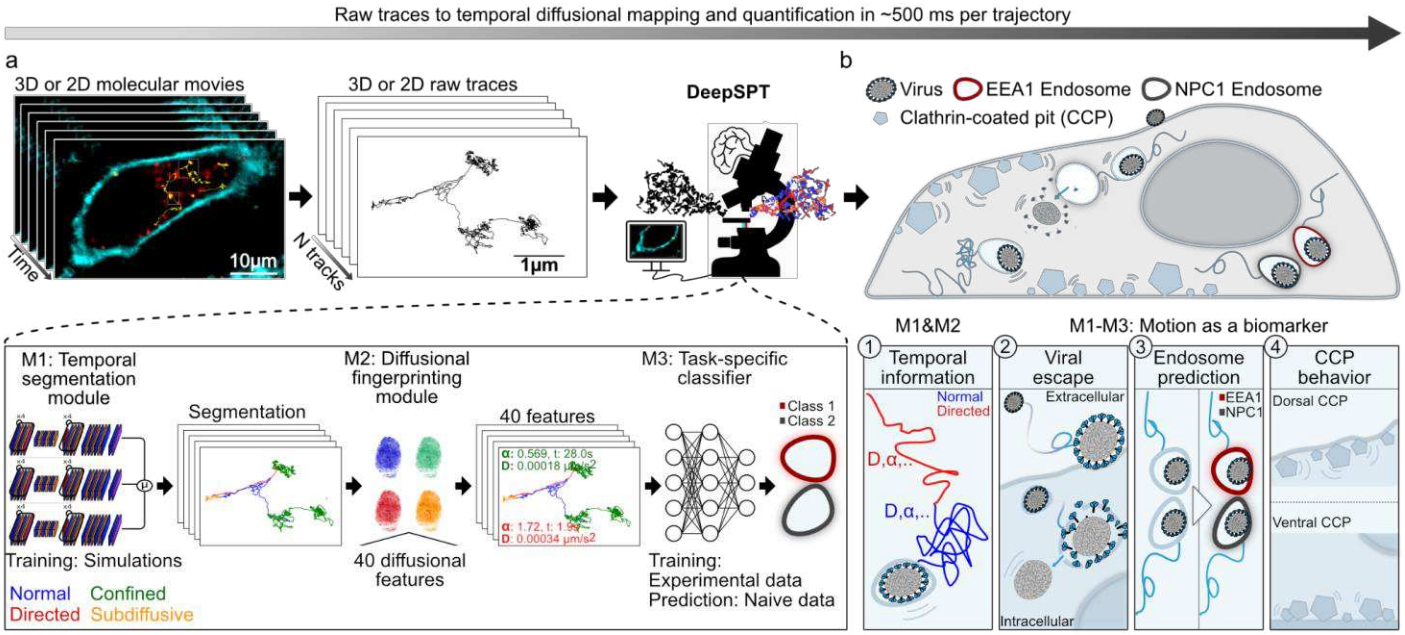

**Fig. 1 | DeepSPT, an agnostic, automated approach for extraction of time-dependent behavior in dynamic systems**. **a,** Schematic representation of the DeepSPT pipeline: 2D or 3D molecular movies from fluorescence microscopy imaging produce a set of x, y, (z), t, localizations for each particle yielding a dataset of single particle trajectories. These trajectories are directly fed to DeepSPT consisting of a temporal behavior segmentation module (M1), diffusional fingerprinting module (M2) and a task-specific classifier (M3). The modules of DeepSPT appear in the zoom-in. Firstly, the temporal segmentation module classifies per time point the diffusional behavior (normal, directed, confined or subdiffusive). Secondly, tracks segmented into diffusional behaviors are quantified by multiple diffusional descriptors by the diffusional fingerprinting module. Thirdly, a task-specific classifier trained utilizing the temporal information and the diffusional fingerprints for each track to learn a problem of interest, e.g., identification of endosomal identity based on diffusional behavior of cargo. The entire DeepSPT pipeline has a computational time of ~500ms per trajectory. **b,** Schematic illustration of selected biological applications enabled by DeepSPT pipeline: 1) Temporal diffusional behavior segmentation, analysis and quantification. 2-4) Applications of DeepSPT to uncover biological insights, based exclusively on diffusional behavior variation. 2) Time point identification of biological events such as detection of viral escape into the cytosol. 3) Prediction of endosomal identity directly using endosomal motion or solely from movement of their cargo. 4) Predicting cellular localization of clathrin-coated pits.

### RESULTS

#### DeepSPT

DeepSPT is a deep learning framework, encompassing three sequentially connected modules: A temporal behavior segmentation module; a diffusional fingerprinting module; and a task-specific downstream classifier module (Fig. 1a). The first two modules are universal, applicable directly to any trajectory dataset characterized by x, y, (z) and t coordinates across diverse biological systems. The final module capitalizes on experimental data to learn a task that is specific to the system under investigation.

The temporal behavior segmentation module transforms single particle trajectories into sub-segments characterized by distinct diffusional behaviors, processing input directly from x, y, (z), and t coordinates using an ensemble of fully convolutional networks (see Methods). Alongside the predicted diffusional behavior, each time point in the trajectory is assigned a probability estimate for each type of diffusion identified. This study focuses on the four types of diffusional behaviors predominantly reported in biological system[16,25,32,40–42]: 1) Normal diffusion, typifying unhindered random motion; 2) directed motion, as commonly exhibited by molecular motors; 3) confined motion, characterizing limited spaces with reflective boundaries, such as small membranes structures; and 4) subdiffusive motion, indicative of more restrained movement, commonly observed in densely populated cytosolic environments. The module's training utilized an extensive dataset comprising 900,000 trajectories, exhibiting broadly distributed diffusional properties; this encompasses variations spanning four orders of magnitude in diffusional coefficients, diverse trace durations, varying localization errors, and trajectories displaying multiple, random length diffusional behaviors throughout their lifespan (see Methods). This extensive training set expands DeepSPT's adaptability across different biological systems and experimental conditions. It is important to note that DeepSPT can be trained to recognize other diffusional attributes and diverse motion types, or to simply predict a uniform global diffusional state in cases of homogenous motion.

The diffusional fingerprinting module within DeepSPT, transforms each identified segment of diffusional behavior into a comprehensive set of 40 descriptive diffusional features, not just encompassing but also expanding upon those enunciated by Pinholt et al[21]. This module serves dual purposes: it facilitates statistical quantification of individual behavior segments for user interpretation; and it generates feature representations crucial for downstream classification tasks (see Methods).

The task-specific downstream classification module of DeepSPT trains and predicts directly on experimental data, which has been transformed to a combined feature set by the temporal and diffusional fingerprinting modules. This module outputs class probability estimates solely utilizing diffusional characteristics for any domain. This is exemplified by predicting important time points during the initial phases of rotavirus infection, differentiating early and late endosomes, and by locating clathrin-coated pits and vesicles to the dorsal or ventral membranes of a cell (Fig. 1b).

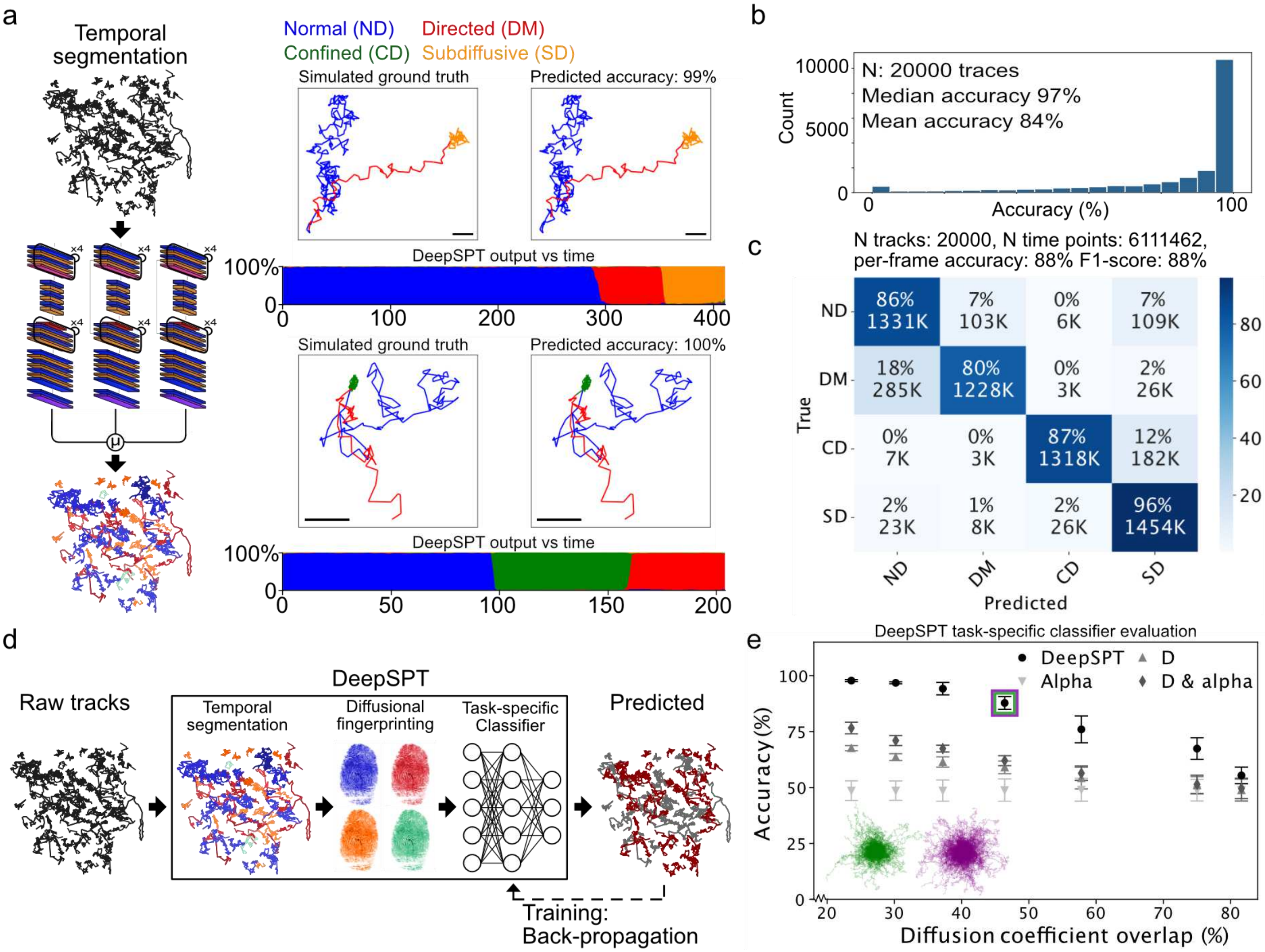

**Fig. 2 | Evaluation of DeepSPT's temporal behavior segmentation. a,** Illustration of the temporal segmentation module of DeepSPT along with two examples of DeepSPT prediction on simulated trajectories with heterogeneous diffusion. For representative 3D examples of predictions: Left: 2D projection simulated ground truth color coded to underlying diffusional behavior. Right: Trajectory color coded to DeepSPT's predictions. Scale bar 500 nm. Bottom: Uncertainty calibrated probability estimates (DeepSPT output vs. time) for each modeled diffusion type per time point, providing transparency into model certainty associated with a given prediction (see Supplementary fig. 3 for uncertainty calibration by temperature scaling). **b,** Histogram of the accuracies associated with each individual 3D trajectory in the test set (N traces: 20.000, N time points: 6111462) and population descriptive statistics such as a median accuracy of 96% (see methods for test set specification). **c,** Confusion matrix based on all predictions (N time points: 6111462) within the 20,000 3D test set trajectories in **b** totaling >6M individual time point predictions. Diagonal entries are correct predictions and off-diagonal indicates confused classes. Each entry reports the absolute number of predictions (K=1000) and its normalization to the actual number of labels in the given class. F1 score of 88% shows DeepSPT to accurately segment and classify heterogeneous diffusion. **d,** Illustration of the DeepSPT classification pipeline. Each raw track is temporally segmented to each of the four diffusional behavior by the segmentation module, transformed into descriptive features by the diffusional fingerprinting module which combines to a unique feature set of temporal and diffusional features which subsequently is fed to a task-specific downstream classifier. **e,** Benchmarking of the DeepSPT classification pipeline against a classifier using MSD features: Diffusion coefficient (D), the anomalous diffusion exponent term (alpha), or both (D & alpha) on simulated data of two classes of trajectories with overlapping diffusional properties (see Methods). Classification accuracy is evaluated at incrementing degrees of overlap in the instantaneous diffusion coefficients. Purple and green trajectories depict trajectories at ~45% overlap in diffusion coefficients indicated by the purple and green boxes. DeepSPT significantly outperforms all three MSD features up to 75% overlap in diffusion coefficient (all p-values < 0.001 using two-sided Welch t-test, N=5 per condition) and at 82% overlap DeepSPT significantly outperforms D and alpha (all p-values < 0.05).

### Rapid, Automated Analysis of Temporal Diffusional Behavior

To demonstrate the effectiveness and generalizability of the temporal segmentation capabilities of DeepSPT, we employed three distinct evaluation schemes. First, we used a holdout scheme to assess performance on trajectories withheld during training (Fig. 2a-c). Second, we tested the model's generalizability using simulated trajectories with a wider distribution in the values of diffusion parameters than those used during training. Third, we compared DeepSPT's performance against existing state-of-the-art temporal segmentation algorithms.

In the holdout validation, we assessed the temporal segmentation on a test set comprising 20,000 simulated trajectories, of which 80% exhibited heterogeneous motion and 20% showed homogeneous motion (see Methods). The trajectories spanned a broad range of diffusional parameters and four motion types (Fig.2a, see Methods, Supplementary fig. 4). DeepSPT not only accurately identified temporal changepoints (Fig. 2a) but also yielded time-resolved probability estimates that may serve as an adjustable post-processing parameter (Fig. 2a, DeepSPT output vs. time). We calibrated these probability estimates using temperature scaling[43] (Supplementary fig. 3) to enhance reliability and mitigate overconfidence,

Quantification of DeepSPT's classification performance revealed a median accuracy of 96% per trace and 84% mean accuracy per frame for all four motion types (Fig. 2b,c, and Supplementary fig. 1). The model achieved 91% mean accuracy for three motion types—normal, directed, and confined/subdiffusive—and 97% for two motion types normal/directed versus confined/subdiffusive motion types (Supplementary fig. 1); and 91% for homogeneous motion (Supplementary fig. 2). DeepSPT achieved an F1 value of 88% for both 3D and 2D data sets (Fig. 2c, Supplementary fig. 1). In all cases it has an inference time of less than 40 ms per trajectory. Subdiffusive motion was classified with 96% accuracy, directed motion with 80%, normal and confined motion with 86% and 87% accuracy, respectively. Minimal confusion existed between dissimilar motion types, highlighting DeepSPT's capability to differentiate between restricted and free motion types. This strength became more apparent when the model was tasked with identifying fewer motion categories (Supplementary fig. 1). Robust performance of DeepSPT was further confirmed across a variety of diffusional properties, track durations, and localization errors, even for parameter ranges not included in the training set. DeepSPT excelled for traces longer than 20 frames and localization errors equal or smaller than the actual diffusional step lengths (Supplementary fig. 8-10)., demonstrating its adaptability to various experimental setups.

We benchmarked DeepSPT’s ability to segment heterogeneous diffusion against a high-performing LSTM-based method[31] and the widely used rolling MSD approach[14–17,22] (Fig. 1c). We focused on these methods over others, such as HMM[24–27], because they do not require large variations in step lengths to detect changes in diffusional behavior (see Supplementary fig. 11,12). Each method was tested on 2D trajectories, given the LSTM-based technique's 2D limitation. The LSTM-based method achieved classification accuracies of 44%, 58%, and 72%, outperforming the rolling MSD's 34%, 51%, and 65% for four, three, and two diffusional behaviors, respectively. DeepSPT, in contrast, attained accuracies of 88%, 91%, and 97% for the same categories, outperforming current state-of-the-art and highlighting its improved competence in agnostic segmentation and classification of heterogeneous diffusion.

We then qualitatively evaluated DeepSPT on 2D experimental data sets (Supplementary fig. 6,7). Specifically, for human insulin we labeled with Atto-655 and recorded its spatiotemporal localization in HeLa cells using 2D using live-cell spinning disk confocal fluorescence microscopy (see Methods). Using DeepSPT we report insulin intracellular transport mainly exhibited subdiffusive behavior but included segments of directed motion. The directed motion aligns with motor-protein diffusion patterns indicative of active cellular trafficking, establishing DeepSPT as a potential tool for studying transport mechanisms across diverse experimental contexts.

Classification of biomolecular identity requires an addition to temporal segmentation. We combine all modules of DeepSPT to demonstrate its capabilities to leverage subtle diffusional variations to classify heterogeneous behavior. The integration of DeepSPT's segmentation and fingerprinting modules allows for the transformation of any trajectory into a feature representation containing both temporal and diffusional features, which can then be fed to a downstream classifier (Fig. 2d). To demonstrate the descriptive power of DeepSPT’s integrated approach, we assessed the classification performance of DeepSPT on two classes of simulated trajectories (1000 tracks) with overlapping diffusional properties (see Methods). Keeping all diffusional features except the diffusion coefficient constant, we evaluated the classification accuracy by stratified 5-fold cross-validation for varying degrees of overlap in diffusion coefficients between the classes (see Methods). DeepSPT achieved up to 98% accuracy and maintained 76% accuracy even when the overlap in diffusion coefficients was around 57%. This performance significantly (by Welch t-test, fig. 2e) outperformed that of basic MSD features, which attained accuracies ranging from approximately 49% to 76% (Fig. 2e). Such robust classification highlights DeepSPT's ability to discern and exploit subtle differences in diffusional properties.

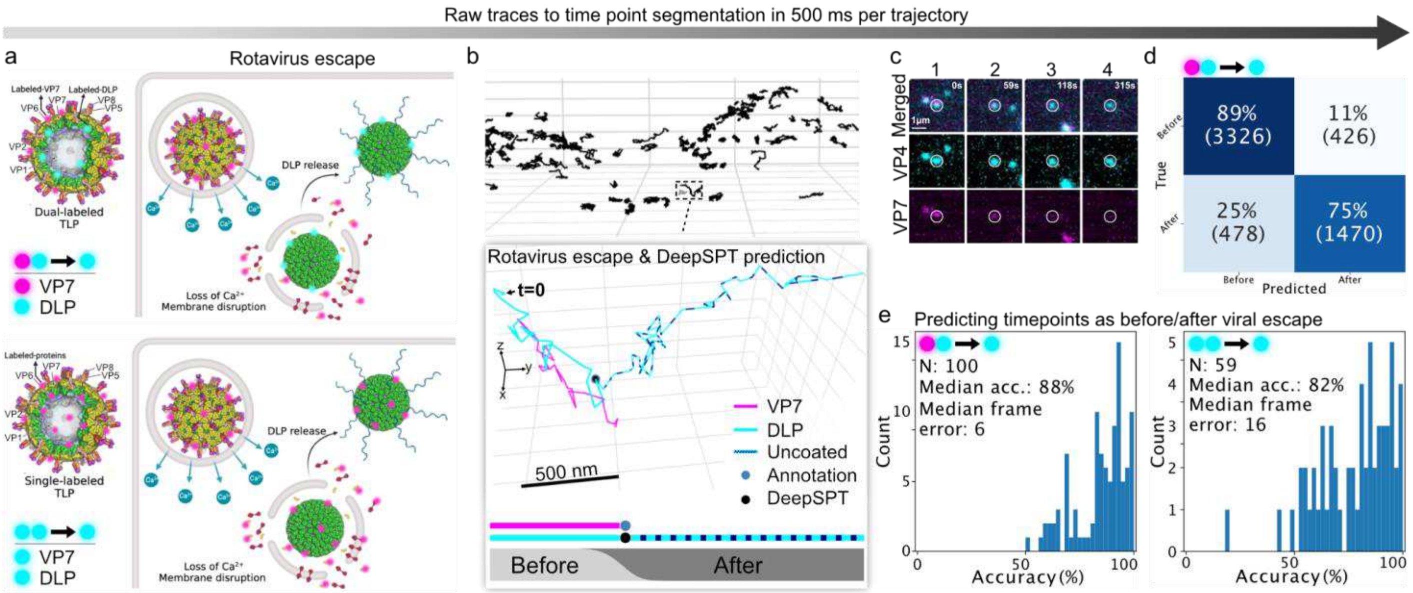

**Fig. 3 | Rapid and precise classification of rotavirus uncoating by DeepSPT based exclusively on diffusional behavior. a,** Schematic illustration of typical stages of rotavirus cell-entry pathway from interactions at the plasma membrane, membrane engulfment, membrane permeabilization, calcium-dependent uncoating and escape to the cytosol where RNA production can begin. Top and bottom panel display the two experimental approaches used for single rotavirus tracking: Top, Dual-labeled by recombinant construction of rotavirus with fluorescently tagged DLP and VP7. Bottom, monochromatic, chemical labeling of free lysines by Atto560. **b,** 3D tracks of individual rotavirus particles in a single cell acquired by live-cell lattice light sheet microscopy (LLSM). Zoom-in: Example of rotavirus by parallel multicolor imaging of DLP and VP7 (see Methods). The time point for loss of VP7 signal, indicative of uncoating and viral escape (blue dot), is correctly identified by DeepSPT (black dot). Bottom Insets: 1D representation of DLP and VP7 signal with annotations for loss of VP7 and DeepSPT prediction. Softmax output, providing time-resolved probability estimates of "before uncoating" and "after uncoating". **c,** Sum intensity projections of the 3D live-cell LLSM raw data from a region of interest surrounding the track in the zoom-in. The insets contain parallel imaging of DLP (cyan) and VP7 (magenta). Numbered columns showing different observed stages of the virus's lifetime for DLP and VP7 from colocalization to uncoating. **d,** Confusion matrix displaying DeepSPT classification performance of predicting time points as "before uncoating" or "after uncoating" as compared to ground truth colocalization analysis, entries normalized to true labels for dual-labeled rotavirus (see Methods) (top left: True before, top right: False after, bottom left: False before, bottom right: True after). **e-f,** Histogram of DeepSPT classification accuracies as percentage of time points correctly predicted "before uncoating" or "after uncoating" in individual tracks. **e**, Dual-labeled rotavirus showing median accuracy 88% (100 tracks, N=1 coverslip experiments, 4 movies). **f,** monochromatically labeled rotavirus showing median accuracy 82% (59 tracks, N=5 coverslip experiments, 13 movies). DeepSPT requires 500 ms processing time per trajectory to transit from raw trajectories to unique feature representations to classification of time points (classification time is ~1 millisecond), thus, accelerating the analysis by a minimum of 4 orders of magnitude as compared to manual annotations.

### DeepSPT accelerates detection of viral escape using motion as a marker.

We validated the operational efficacy of DeepSPT to extract information from 3D live-cell SPT data of rotavirus (Fig. 3a, see Methods). The entry process of rotavirus into cells, the first step for infection, involves glycolipid-mediated membrane association of the virus, vesicular engulfment and internalization, virus-initiated membrane permeabilization, calcium-dependent uncoating of outer proteins, membrane disruption, and cytosolic delivery of the viral genome for subsequent RNA production (Fig. 3a)[44]. Previous single-particle tracking in BSC-1 cells using confocal imaging indicated that the uncoating step correlates with a change in diffusional behavior, hinting motion as a potential marker of biological behavior[44,45].

To test DeepSPT's capacity to detect the uncoating and cytosolic delivery events using only motion, we used 3D live-cell lattice light sheet microscopy[5,46] (LLSM ) to image cell entry of reconstituted rotavirus[44,47] labeled with either: Atto560 on VP7, an outer shell protein, and Atto642 on DLP, the double layered particle; or with Atto560 on the entire virus including both VP7 and DLP (see Fig. 3a,b). Trajectories captured via LLSM (see Fig. 3b) underwent temporal segmentation and diffusional fingerprinting using DeepSPT's modules in rolling windows for sequential representation (see Methods). These processed trajectories were then classified as either "before uncoat" or "after uncoat" through a sequence-to-sequence based model, transforming coordinates in time into time-resolved predictions, serving as the task-specific classifier (see Methods, Supplementary movie 1 & 2). The ground truth for uncoating events for dual-labeled rotavirus was established by determining the extent of colocalization of differentially labeled DLP and VP7 (see Methods). A representative example of rotavirus uncoating alongside DeepSPT's consistent prediction and snapshots of the raw data are shown (Fig. 3b zoom-in and Fig. 3c).

DeepSPT correctly identified 89% of "pre-uncoating" and 75% of "post-uncoating" time points, yielding a mean accuracy of 85% and a median accuracy of 88% across 100 dual-labeled rotavirus trajectories. This high level of accuracy translated to a median error of just 6 frames in determining the uncoating time point (Fig. 3d,e). Unlike traditional methods, which often require manual analysis taking several minutes to hours per trajectory, DeepSPT automated the identification process, reducing the time to milliseconds per viral trajectory offering massive acceleration and minimizing any human bias.

Importantly, DeepSPT outputs these predictions, in 500 ms per trajectory, based solely on the motion captured in the DLP trajectories, rendering the secondary VP7 channel redundant (Supplementary movie 1 & 2). When tested on rotavirus labeled with the same fluorophore on both DLP and VP7 (see Methods), DeepSPT exhibited similar performance, achieving a median accuracy of 82% and a mean accuracy of 78% (see Methods, Fig. 3f). It's worth noting that it required labor-intensive (~8 working days) manual annotation to acquire the ground truth annotations based on intensity loss of the 560 nm channel (see Supplementary fig. 13) while taking less than a minute for DeepSPT. By using motion as a marker for viral uncoating, DeepSPT simplifies experimental design and preparation, avoiding the need for constructing dual-labeled viruses. Thus DeepSPT frees up one of the 2-3 available imaging channels, thereby increasing the information content in fluorescence microscopy experiments. To the best of our knowledge, these results constitute the first instance of detecting viral escape into the cytosol based solely on motion, and without the need for multicolor labeling.

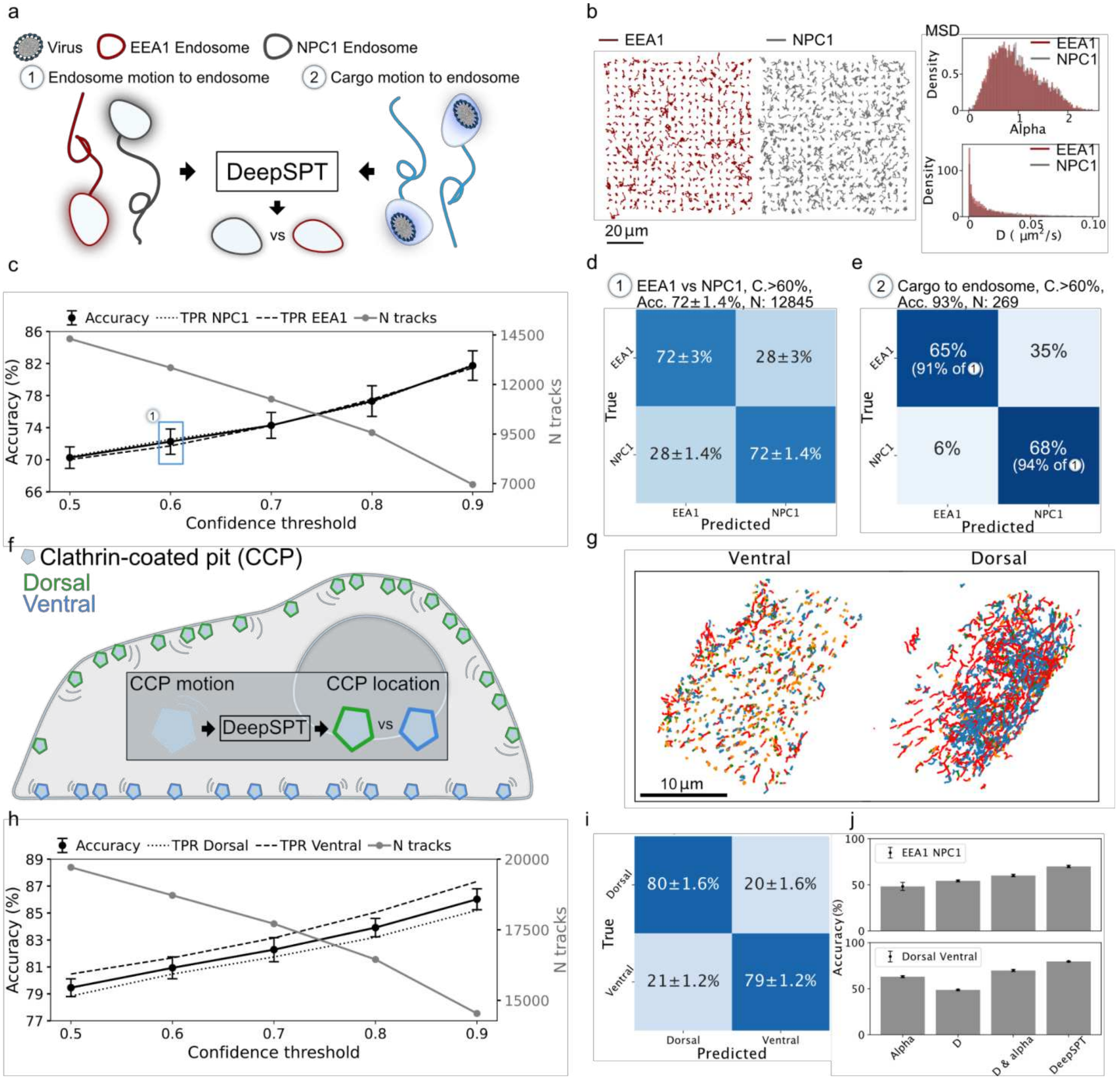

**Fig. 4 | Prediction of endosomal identity and AP2 cellular localization based exclusively on temporal variations in diffusional behavior. a,** Illustration of endosomal identity prediction solely on the diffusional properties of the endosomal marker or by the motion of endosomal cargo. **b,** Left 2D projection of randomly sampled trajectories of EEA1-mScarlett- (dark red) and

NPC1-Halo-JFX646- (gray) positive endosomes acquired by 3D LLSM revealing visually similar trajectories. Right: Distribution of alpha anomalous coefficient (top) and diffusion coefficient (bottom) for EEA1-positive (dark red) and NPC1-positive (gray) displaying practically indiscernible distributions. **c,** Twin axes plot of accuracy and true positive rate (TPR) (left) as well as number of tracks (right) versus confidence threshold (see Methods) of the full classification pipeline of DeepSPT (Fig. 2d). Increasing the confidence threshold enhances accuracy and reduces the number of accepted traces. **d,** Confusion matrices of DeepSPT classification accuracy on prediction of EEA1-positive vs NPC1-positive compartments showing accuracy of 72+-1.4% with a confidence threshold at 60% (top left: True EEA1, top right: False NPC1, bottom left: False EEA1, bottom right: True NPC1). Data is from (N=4 coverslip experiments, 35 movies). **e,** Confusion matrix of DeepSPT classification accuracy on prediction of EEA1-positive vs NPC1-positive compartments now using trajectories of their cargo, i.e., rotavirus with the confidence threshold at 60%. Results in parenthesis show accuracies normalized to results of (d) to compare to the direct prediction accuracy of endosomes. Data consists of 269 tracks (N=12 coverslip experiments, 44 movies). **f,** Illustration of prediction of AP2 complexes' cellular localization by DeepSPT based exclusively on their diffusional behavior. **g,** 2D projections of AP2 trajectories below and above 500 nm from the coverslip. All trajectories spending >20% of their lifetime above the 500 nm are considered dorsal, while the rest are considered ventral. Trajectories are color-coded by DeepSPT segmentation of diffusional behavior. **h,** Twin axes plot of accuracy (left) and true positive rate (TPR) as well as number of tracks (right) versus confidence threshold (see Methods) of the full classification pipeline of DeepSPT for AP2 data. **i,** Confusion matrices of DeepSPT classification accuracy on prediction of AP2 showing accuracy of 79.5+-0.6% at 50% confidence threshold. Data consists of 19712 tracks from (N=5 coverslip experiments, 13 movies). **j,** Benchmark of using DeepSPT versus conventional metrics based on MSD features: Diffusion coefficient (D) or the anomalous diffusion exponent term (alpha) for both EEA1-positive compartments vs NPC1-positive (EEA1NPC1) and dorsal AP2 vs ventral AP2. DeepSPT significantly outperforms common MSD analysis (all p-values $< 0.00001$ using two-sided Welch t-test, N=10 per condition).

#### DeepSPT enables label-minimal colocalization analysis and identifies cellular localization through motion

The capacity to identify biomolecular identity, colocalization partners or to infer subcellular localization based solely on diffusional properties could minimize the need for multicolor imaging and the labor-intensive efforts associated with the creation of cell lines expressing the relevant fluorescent cellular markers. Early and late endosomes for example are difficult to differentiate without multicolour labeling as they might appear to exhibit similar dimensions, are distributed with similar spatial density, and display nearly identical diffusion coefficients[49–51]. Traditionally, their identification requires labeling of each endosomal type through antibodies specific for endogenous protein markers enriched in a given type of endosome, or by ectopic expression of these markers. Based on multicolour labeling, efforts have been made using population-based multiparametric image analysis of internalized cargo distribution and compartment morphology in fixed samples to deduce general principles of the endocytic machinery[48].

Here we assess whether DeepSPT can determine endosomal identity based solely on diffusional characteristics, reducing the need for multicolour labeling (Fig. 4a). We used two-color live-cell LLSM to track early endosomes endogenously tagged by gene editing with (EEA1-mScarlett) and late endosomes tagged with (NPC1-Halo-JFX646) (Supplementary movie 3). Their trajectories display indistinguishable diffusion coefficients and alpha values (Fig. 4b), as well as similar diffusional behavior propensities (Supplementary fig. 14) challenging endosomal identity prediction. To address this issue, we introduced a decision confidence threshold, requiring the classifier's probability estimate to surpass this threshold for prediction acceptance[35]. In a 10-fold stratified cross-validation scheme with varying decision confidence thresholds, DeepSPT achieved accuracies ranging from 70±1.3% to 82±1.8% in classifying EEA1-positive from NPC1-positive compartments. Increasing the confidence threshold improved accuracy but reduced the number of accepted tracks (Fig. 4c). At a 60% confidence threshold, DeepSPT identified endosomal types with an accuracy of 72±1.4% (Fig. 4d), and a recall of 72±3% for EEA1-positive compartments and 72±1.4% for NPC1-positive compartments (Fig. 4d). DeepSPT significantly outperformed the commonly used MSD analysis that reached accuracies of 48±4%, 55±1.6 and 60±1.4% (Supplementary fig. 15,16) in endosomal classification by using the variation in alpha values, variation in the diffusion coefficient (D), or combining both alpha and D, respectfully. DeepSPT, solely using the diffusion traits of endosomal cargo, achieved 91% and 94% of the recall observed in direct prediction of EEA1-positive and NPC1-positive compartments, respectively (Fig. 4e). The ability of DeepSPT to differentiate early from late endosomes based solely on their motion or that of their cargo, accelerates data acquisition and analysis while minimizing potential perturbations or/and the need for multicolour tagging.

To assess the universality of DeepSPT to infer identity, we applied it to a new dataset of single-particle trajectories of the assembly of clathrin-coated pits and coated vesicles forming at the cell surface. The dynamic assembly and intracellular location of these structures was obtained by tracking the clathrin AP2 adaptor complex, gene-edited at its sigma subunit with eGFP, using 3D live LLSM. A 2D projection of the acquired AP2 trajectories qualitatively indicated that the diffusional properties of AP2 were correlated with cellular location[52] —dorsal versus ventral cell surface (Fig. 4f,g). This correlation was quantitatively confirmed by DeepSPT's temporal segmentation of the 3D traces (Supplementary Fig. 14). DeepSPT accurately predicted the cellular location of AP2 in a 10-fold cross-validation scheme, yielding accuracies that varied from 79.5±0.6% to 86.0±0.8% at different confidence thresholds (Fig. 4h). Without applying any confidence filter (i.e., a 50% threshold), DeepSPT classified the cellular location of AP2 with recalls of approximately 80% for both classes (Fig. 4i). In contrast, pinpointing the cellular location of AP2 using MSD features reached accuracies of 62.5±1.8% 48.7±0.8% and 70±1.3%, with a maximum recall for dorsal tracks of 60±2% (Fig. 4g, Supplementary fig. 15,16). Subtle diffusional variations across systems, while missed by common tools, are utilized by DeepSPT to precisely output biological information in complex systems. DeepSPT achieves this across various biological contexts, imaging protocols, and experimental conditions.

#### Discussion

The diffusion of biomolecules within cells exhibits both spatial and temporal heterogeneity and varies across biological systems and functionalities but extracting quantitative temporal information from live-cell imaging is currently an analytical bottleneck and often relies on system-specific analysis or even manual annotation. DeepSPT overcomes this bottleneck by providing a universal framework to transition from raw trajectories to quantitative temporal information rapidly precisely and with minimal human intervention both 2D and 3D diffusion. Trained on trajectories with broadly distributed diffusional properties, DeepSPT consistently outperformed existing state-of-the-art toolboxes both in segmenting and classifying diverse heterogeneous diffusional behaviors, both in simulated and experimental data. The implementation of uncertainty-calibrated probability estimates enhances the transparency of DeepSPT's output, enabling users with limited a priori knowledge of the biological system to gauge model certainty. The minimal requirement for human intervention highlights DeepSPT's potential to enhance both the reproducibility and robustness of conclusions across different laboratories. Being open-source and freely available to the public, allows future users to perform customized analyses according to individual research needs.

The precise temporal segmentation combined with the comprehensive quantification of diffusional properties of DeepSPT, coupled with its trained downstream classifier, facilitate rapid prediction of viral uncoating events—achieving results in seconds as opposed to weeks as required for manual annotation. This four orders of magnitude acceleration, not only marks the first deep-learning-assisted identification of viral uncoating but also shifts the bottleneck in single-particle discoveries from data analysis to data acquisition. It even introduces the potential for virtually real-time analysis of early stages of viral infection.

Subtle diffusional variations in 2D or 3D, while missed by common tools, are utilized by DeepSPT to precisely output biological information in complex systems across various biological contexts, imaging protocols, and experimental conditions. For example, DeepSPT discerned EEA1-positive from NPC1-positive compartments solely based on their respective 3D diffusional characteristics, or that of their cargo with accuracies of 72% significantly outperforming the commonly used MSD analysis that reached accuracies of 50-60%. These findings prompt further mechanistic studies to explore whether divergent diffusional behaviors stem from distinct external interaction partners, inherent physical differences between endosomal compartments, or other variables. DeepSPT similarly pinpointed the cellular location of AP2 on 3D data with an accuracy of 80%, significantly outperforming common analysis reaching ~50-70% accuracy. The distinct diffusional behaviors of AP2 highlighted the importance of careful selection in imaging setups. Applied on 2D data of insulin internalization DeepSPT found insulin mainly exhibits subdiffusive behavior but included segments of directed motion indicative of active transport. DeepSPT's ability to accurately quantify heterogeneous behaviors in both 2D and 3D, across diverse biological systems and under varying experimental and imaging conditions, attests to its utility as a universal platform for characterizing heterogeneous diffusion across systems.

DeepSPT's capacity for predicting viral uncoating events, identifying endosomal types, and discerning colocalization partners and cellular localization solely based on diffusion extends the traditional structure-to-function paradigm in proteins to a novel motion-to-function paradigm. This suggests that, alongside structure[9,53–55], motion can also serve as an indicator of both function and identity. This development opens avenues for employing motion as a biomarker and for label-minimal analyses—effectively substituting fluorescent labels with temporal diffusional analysis. Such a shift could simplify experimental design and reduce preparation time, or potentially enrich experiments by reallocating redundant fluorescent markers for other applications.

Widespread implementation of DeepSPT across laboratories could facilitate the creation of comprehensive libraries detailing characteristic movements of cells, subcellular structures and biomolecules. An open-source diffusional library of this kind would offer a new instrument for the scientific community, aiding in the exploration of 4D cell biology through temporal diffusional behavior.

**Author contributions** J.K.H., N.S.H. and T.K. wrote the paper with feedback from all the authors. J.K.H. performed all computational work with input from T.K. W.B. and N.S.H. M.D.S. performed all rotavirus preparation. A.S., R.D.C.C. and G.S. performed LLSM imaging with the presence of JKH at T.K. laboratory at Harvard Medical School. A.J.J., and S.B. performed SDCM imaging and insulin assays at N.S.H. laboratory at University of Copenhagen. J.K.H. and N.S.H. conceived the project idea. N.S.H. had the overall project management with tight interactions with T.K.

**Acknowledgement**
We thank members of our laboratories for help and encouragement. We thank Narendra Kumar Mishra and Professor Knud Jørgen Jensen for discussion concerning the preparation and use of insulin for our experiments. This work was funded by the Villum Foundation by being part of BioNEC (grant 18333) to J.K.H. and N.S.H., the Novo Nordisk foundation challenge center for Optimised Oligo escape (T.K., co-PI), the center for 4D cellular dynamics N.S.H. is affiliated with The Novo Nordisk Foundation Center for Protein Research (CPR) funded by a generous donation from the Novo Nordisk Foundation (grant no. NNF14CC0001). J.K.H., A.J.J., S.B., and N.S.H. are members of the Integrative Structural Biology Cluster (ISBUC) at the University of Copenhagen. VILLUM FONDEN (40516) for W.B. and N.S.H. The Novo Nordisk Foundation Center for Basic Machine Learning Research in Life Science (NNF20OC0062606) for W.B. T.K. acknowledges support from NIH Maximizing Investigators' Research Award (MIRA) GM130386, NIH Grant AI163019, 1R01, IONIS Pharmaceuticals. M.D.S was supported by NIH/NCI CA13202 grant to S.C.H. Figure 3a was created with BioRender.com.

**Data and code availability**
All raw data, treated data, and python code can be found in University of Copenhagen repository:

https://erda.ku.dk/archives/d88752311f3730f6e0f93c3aa3b0da50/published-archive.html
All code will freely available upon publication on GitHub as well.

### Methods

**DeepSPT's diffusional fingerprinting module.** Encompassing and expanding on the work of Pinholt et al[21], a recent study providing a set of diffusional metrics to transform single particle trajectories into fixed length representations of interpretable features functioning as unique identifiers, i.e., diffusional fingerprints, this work both extends the number of descriptive features of diffusional behavior from 17 to 40 and importantly provides temporal features and sequential representations to enable time-resolved predictions. This is outlined in the following sections.

**Confinement radius (r) and directed velocity (v).** As DeepSPT allows accurate diffusional behavior segmentation the possibility of using MSD equations adapted for the specific motion types such as $MSD = r^2(1 - A_1 e^{\frac{-2A_2 dDt}{r^2}}) + offset$, where r is the confinement radius, $A_1$ and $A_2$ are shape parameters, d is the number of dimension and $MSD = 2dDt^2 + v^2t^2 + offset$ for confined and directed, respectively[32]. The offset in MSD analysis corresponds to the constant contribution to MSD from localization error. Thus, the velocity term of directed motion and the confinement radius for confined motion can be extracted for whole trajectories or for subtracks.

**Directionality analysis using vector dot products.** The dot product (DP) between two vectors informs of the angle between them and the product of their magnitudes, specifically for normalized vectors the dot product returns the cosine of the angle [-1,1] with 1 for parallel vectors, 0 for orthogonal and -1 for anti-parallel. For a given trajectory the two vectors ([$p_0$,$p_1$] and [$p_1$,$p_2$]) formed by the three consecutive coordinates; $p_0$, $p_1$, $p_2$ can be used to compute the dot product along the trajectory, to investigate persistence in directionality, for matching the length of the original trajectory 2 zeros are added to start the dot product series. Three strategies are used to aggregate the per-frame dot product series into a single value to complement the diffusional fingerprinting. 1. Averaging (MeanDP) to show any average tendency in directionality, where ~0 is to be expected for normal diffusion, >0 for directed and <0 for subdiffusive. 2. Autocorrelation (corrDP) to investigate if consecutive vectors in general tend to have direction persistence, specifically, the percentage of successive occurrences both being positive or negative is computed. 3. Sign analysis (AvgSignDP) counts the percentage of vector dot product signs being positive.

**Additional step length descriptive statistics.** Step lengths contain much information on single particle tracks, here we add additional descriptive statistics such as minimum (MinSL), maximum (MaxSL), broadness of the step length distribution (BroadSL), i.e., MaxSL-MinSL, coefficient of variation of the step length distribution is the ratio between standard deviation and mean, which measures the variability of the distribution in relation to the mean. Arrested fraction and fast-moving fraction are system-specific but computed as the percentage of steps under 0.1 microns and above 0.4 microns, respectively. Calculation of the instantaneous diffusion coefficient was implemented using the mean square displacement between adjacent positions: $MSD / 2dDt$.

**Volume/area of trajectories.** The area or volume (referred to as volume for consistency) of 2D or 3D trajectories is the volume of the convex hull enclosing the trajectory xy(z)-coordinates in 2D or 3D, respectively. The volume of a trajectory or a subtrack is a direct reflection of the trajectory shape and holds information on the amount of volume explored by the trajectory and indirectly the morphology of the explored region. Therefore, the volume may be used to identify restricted particles versus more freely moving and provide a metric for the volume of confinement. Computed using the Python package SciPy[56].

**Temporal features.** To inform a classifier of temporal variation in trajectories the temporal segmentation was condensed into unique features. Four features constructed from the percentage of time spent as either normal diffusion, directed motion, confined diffusion or subdiffusive and a feature from the number of changes in diffusion. To inform on the history of diffusional changes the sequence of diffusional behaviors was encoded as normal diffusion: 0, directed motion: 1, confined diffusion: 4, and subdiffusion: 6, e.g., 0146 for sampling each behavior once starting with normal diffusion. Encoding values were purposely chosen to encode similar motion types to similar values while encoding unique distances between values for each motion type. Six features were constructed from this encoding: Mean and median informing on most likely motion type. Maximum and minimum informing on sampled motion. Standard deviation and median of distances between adjacent sequence values to inform on the changes and similarities of sampled motion types.

**DeepSPT's temporal segmentation module**. The temporal segmentation module within DeepSPT consists of an ensemble of three U-Nets [57] providing end-to-end transformation from raw trajectories to trajectory segmentation. Each U-Net model is trained on a data set of 300000 simulated trajectories as described in the simulation section with 80% heterogeneous motion and 20% homogeneous. The end-to-end architecture of the well-known U-Net can be seen as a down-sampling by an encoder network, an up-sampling by a decoder network followed by a classifier. Individual U-Nets contain 1-dimensional convolutional layers and max pooling in the encoding network and 1-dimensional convolutional layers and nearest neighbor up-sampling in the decoding network with the encoder and decoder connected by skip connections. The encoding and decoding are directly followed by a series of convolutional layers before an ensemble average SoftMax output. The SoftMax outputs from each model in the ensemble are combined by averaging to produce the final DeepSPT prediction. Specific hyperparameters were found by Optuna's tree-structured Parzen estimator[58] and the best set of hyperparameters, not including ensemble size, were selected based on performance on a test set only used for the hyperparameter search. See our open-source implementation on Github for more detail.

**Temperature scaling.** Neural networks have been shown to be overly confident even in erroneous predictions[43]. Such overconfidence can be mitigated by uncertainty calibration so confidence estimates resemble more the actual ground truth correctness likelihood. Temperature scaling (TS) is one such method, which has been found effective despite its simplicity[43]. TS introduces a constant into the last layer before SoftMax of a classifier and this constant is tuned to minimize negative log likelihood as recent work shows this allows approximation of the actual posterior probability distribution. Measures of uncertainty calibration includes; expected calibration error which effectively is the 1-norm error between perfect calibration and the actual calibration, sharpness which measures the distance between the maximum class probability scores for the k classes and 1 as the perfect classifier would have class probability score of 1 for correct prediction and lastly the negative log-likelihood (NLL), here NLL is reported as the NLL improvement relative to random predictions for a more intuitive metric.

**Stochastic simulation of diffusion.** Tracks are generated with a stochastic diffusion coefficient log-uniformly sampled between 0.0001 to $0.5 \mu m^2/s$. Due to the scale invariance property of diffusion the sampling of D from a broad spectrum equates sampling with varying sized time steps (dt), thus, allows the model to learn the characteristics of diffusional behavior both for varying D and dt. Simulated tracks were generated starting in x=y=0 with lengths uniformly drawn between 5 frames and 600 frames challenging the model to pick up regularities even in shorter traces. Normal, directed and subdiffusion were simulated following Pinholt et al[21] and Kowalek et al[20]. The different parameters for the simulation of these diffusion types were chosen similarly to Wagner et al[32], Pinholt et al[21] and Kowalek et al[20]. Except for three parameters that were made even broader distributed: Alpha, anomalous exponent term measuring motion persistence 0-0.7. Step length to localization error ratio defined as, Q $= \sqrt{\frac{D \cdot dt}{\sigma_{noise}}}$ and Q-directed = $\sqrt{\frac{D \cdot dt + v^2 dt^2}{\sigma_{noise}}}$ with Q and Q-directed uniformly sampled between 1 and 16 and lastly the extent to which active motion affects the diffusion, R = $\frac{v^2 dt}{4D}$ was generated uniformly between 5 and 25. Confined motion also differs from previous work as the confinement radius in this work is independent of track duration reflecting the case where a radius of reflecting boundary area does not grow as the experiment progresses. Instead, we simulated confinements irrespective of the longevity of the tracks. The area or volume of confinement is defined by an ellipse in 2D and an ellipsoid in 3D, allowing any orientation, with the semi-major and semi-minor axes uniformly sampled between 5 nm to 250 nm, for the 3D case the two semi-minor axes chosen to be equilength, thus producing a large range of confinement areas/volumes in any given orientation.

**Generating heterogeneous diffusion**. Trajectories with heterogeneous diffusional behavior were simulated as homogeneous tracks with the addition of sampling random changepoints in diffusional behaviors of up to 4 states. Therefore, a given trajectory was separated into random subtraces with a required minimum length of 5 frames, thus, the length of the trajectory must be larger than the product of changepoints and minimum length. Sampling changepoints randomly inside a trajectory was purposely chosen instead of having the states follow a user-defined Markov model to ensure DeepSPT's decision-making is not influenced by learning an underlying Markov model that does not necessarily resemble anything found in Nature, but rather keeping DeepSPT fully agnostic.

**Simulated test set for evaluation of temporal segmentation.** Evaluation was performed on 20000 withheld simulated trajectories 80% heterogeneous and 20% homogeneous motion with all associated diffusional parameters broadly distributed as described under Stochastic simulation of diffusion.

**Moving simulated diffusion to 3D.** The work of Wagner et al[32], Pinholt et al[21] and Kowalek et al[20] is exclusively focused on diffusion in 2D, where due to our 3D live-cell lattice light sheet experiments and seeking a universal model we are required to extend previous work to 3D. Simulating normal diffusion and subdiffusive motion easily extends to two- and three-dimensional cases as axes of diffusion are independent. Directed motion is in Wagner et al[32], Pinholt et al[21] and Kowalek et al[20] simulated using the cosine and sine to ensure directionality in x- and y-direction respectively with the velocity as a magnitude term, which we extend to the three-dimensional case by considering the unit sphere instead, thus the added velocities become $dx = v \cdot dt \cdot \sin(\phi) \cdot \cos(\theta)$, $dy = v \cdot dt \cdot \sin(\phi) \cdot \sin(\theta)$, $dz = v \cdot dt \cdot \cos(\phi)$, where $\theta$ is the polar angle and $\phi$ is the azimuthal angle.

**Simulation of two populations of heterogeneous diffusion.** Two populations of 500 trajectories each with track durations uniformly sampled between 150 to 200 time points constructed using the aforementioned simulation framework of heterogeneous motion of the four diffusion types (see Methods). Both populations had the step length to localization error ratio uniformly sampled between 6 and 16. Population 1 had active motion ratios uniformly sampled between 5 and 12, subdiffusive motion with alphas uniformly sampled between 0.3 and 0.6. Population 2 had active motion ratios uniformly sampled between 8 and 15, subdiffusive motion with alphas uniformly sampled between 0.4 and 0.7. Otherwise, populations were constructed identically at increments of diffusion coefficients. Diffusion coefficients are log-uniformly sampled between 0.004 $\mu m^2/s$ and

0.0008 $\mu m^2/s$ separating in increments of 0.005 $\mu m^2/s$. After each stochastic simulation of trajectories, the distributions of instantaneous diffusion coefficients were computed as described (see Methods) and overlap in the two distributions computed as the histogram intersection normalized total tracks in one population.

**Time-resolved task-specific downstream classifier using temporal and diffusional features.** Segmentation of rotavirus trajectories into "before uncoating" and "after uncoating" was performed using a sequence-to-sequence model trained on time-resolved diffusional features computed using the temporal segmentation and diffusional fingerprinting module in windows. Raw trajectories can be seen as time series with 3 parallel channels (xyz) per time point; these were transformed into time series of identical length but now 40 channels (one per feature in the temporal segmentation and diffusional fingerprinting modules) utilizing a window of 31 frames centered on each time point in a given trajectory. Ground truth of the uncoating time point for dual-labeled rotavirus was constructed based on loss of colocalization between VP4 and VP7. For single-labeled rotavirus labels are based on manual annotation of the endpoint of intensity drop following loss of VP7 signal (Supplementary fig. 14). Both cases produce binary target time series which are filtered if loss of colocalization is in the first or last frame. The sequence-to-sequence model architecture consists of a bidirectional five-layer gated recurrent unit (GRU) followed by a fully connected feedforward layer. The output length of the GRU is twice the input length due to its bidirectionality, thus split in half and combined by summation to match the input length before the fully connected layer. Before training trajectories are evaluated for similarity by root mean squared distance (RMSD) and trajectories with RMSD less than 0.6 $\mu m$ are grouped as connected components in a graph. Model training is performed using a 10-fold grouped cross validation (validation and test size are 10% each) to ensure similar trajectories are in the same fold while saving the model with highest average recall on validation set.

**Task-specific downstream classifier for prediction exclusively from diffusional characteristics.** In all cases classifiers receive a fixed length representation of trajectories and outputs a probability estimate per class. Fixed length representations are constructed from raw trajectories by using the temporal behavior segmentation and diffusional fingerprinting modules totaling 40 descriptive diffusional features (see Methods). Filtering using a confidence threshold on outputted probability estimates is performed by requiring estimated probabilities to be larger than the given threshold otherwise trajectories are considered to be predicted as "unknown". *Prediction of two simulated populations (Fig. 2e)* consists of a simple logistic regression model from Scikit-learn[59] using "*lbfgs"* as solver with an allowed number of iterations at 10000 evaluated in a 5-fold stratified cross validation with a test size of 10%. *Prediction of EEA1-positive and NPC1-positive compartments (Fig. 4)* is performed using a simple multilayer perceptron model consisting of an input layer (size: 40) and output layer (size: 2) with Softmax activation. Training was performed with random oversampling of the minority class to mitigate majority class bias and evaluation in a 10-fold stratified cross validation. *Prediction of EEA1-positive and NPC1-positive compartments using viral cargo (Fig. 4)* is performed using the same multilayer perceptron model trained in a 90%-10% train-validation split with minority class oversampling, saving the model with highest validation accuracy. *Prediction of cellular localization of AP2 (Fig. 4)* is performed exactly as for EEA1-positive and NPC1-positive compartments.

**Analytical colocalization analysis.** Colocalization is defined based on temporally consistent proximity between trajectories across acquisition channels. For each trajectory of interest in each imaging channel the Lock-step Euclidean distances are computed to trajectories in the secondary channel of interest. A minimum number of consecutive frames within a user-defined search distance threshold is required to be defined as a colocalizing segment. To mitigate spurious peaks in distance between two trajectories interrupting a true colocalization segment a certain number of frames is allowed above the given distance threshold ("forgiveness") a certain number of frames and colocalizing segments on either side will be linked. To simultaneously increase certainty in registered colocalization segments and mitigate registering transient, spurious colocalization multiple filters are added on top of "distthreshold", "min_coloc" and the "foregiveness": Minimum total colocalization length, Minimum average lock-step Euclidean distance and a minimum Pearson correlation between individual coordinates axes were enforced.

*Colocalizing rotavirus and endosomes* were identified using a minimum number of consecutive frames of 5, search distance threshold of 750 nm purposely sat high to account for any interchannel aberration, a forgiveness of 5 frames, Pearson correlation threshold of 0.8, minimum total colocalizing length of 5 frames and a minimum average Lock-step Euclidean distance of 750 nm.

*Colocalizing rotavirus VP4 to VP7 signal* was done by initially correcting chromatic aberrations by identifying long-lived colocalization, computing xyz chromatic shift between VP4 and VP7 signal and shifting all VP7 tracks by their average xyz offset. Initial parameters were: Minimum number of consecutive frames of 5, search distance threshold of 750 nm, forgiveness of 3 frames, Pearson correlation threshold of 0.9, minimum total colocalizing length of 20 frames and a minimum average Lock-step Euclidean distance of 500 nm. Following correction of chromatic offset colocalizing rotavirus VP4 to VP7 signal was performed using: Minimum number of consecutive frames of 3, search distance threshold of 400 nm, forgiveness of 2 frames, Pearson correlation threshold of 0.9, minimum total colocalizing length of 20 frames, and a minimum average Lock-step Euclidean distance of 600 nm.

**Inferring AP2 position relative to coverslip by 3D plane fitting.** AP2 coordinates in a 3D space were obtained by LLSM[5,46]. These coordinates were rotated 30 degree around the y axis of the LLSM imaging direction[5,46] to account for the detection angle of the LLSM by the dot product of coordinates with the standard rotation matrix M = [[cos($\theta$), 0, sin($\theta$)], [0, 1, 0], [-sin($\theta$), 0, cos($\theta$)]], where $\theta$ is the rotation angle in radians. Rotated coordinates point the cell's ventral side in positive z-direction. Utilizing AP2 generally localizes to the plasma membrane, rotated xycoordinates were binned (all bins left-inclusive) in a grid size of 5 μm and for each bin the lowest z-coordinate was extracted representing the most dorsal AP2 coordinates. To account for outliers in the dorsal z-coordinates the Mahalanobis distance (using mean and covariance of all dorsal z-coordinates) was calculated for each dorsal z-coordinates filtering coordinates with distance of 1.8 or above. Resulting dorsal z-coordinates were used to fit parameters of a 3D-plane by minimizing the sum squared distance between dorsal z-coordinates and the plane resulting in an inferred coverslip position. All AP2 coordinates had their distance calculated to the resulting plane.

**Statistical tests.** Comparison of results in fig. 2e and fig 4i were performed using two-sided Welch t-test to evaluate the null hypothesis that the conditions in question have equal means. The Welch t-test was chosen due to its strength as a location test and its robustness to populations with unequal variance and sample sizes.

**Viral and endosomal labeling for LLSM imaging.** Cells with gene-edited early endosomal antigen 1 with Scarlett (EEA1-mScarlett) and Halo-tagged version of the cholesterol transporter Niemann Pick C1 with JFX646 (NPC1-Halo-JFX646) were thawed samples from frozen aliquots generated in the Kirchhausen laboratory by Kang et al[60]. Cells with clathrin adaptor complex, AP2 gene-edited at its sigma subunit with eGFP were samples thawed from Cocucci et al[52]. *For imaging*: SVG-A cells were plated onto coverslips with a diameter of 5 mm inside a 35 mm culture dish at ~50% confluency the day before each experiment. Cells were incubated with 10 μl labeled rotavirus particles at ~40 μg/ mL a MOI of ~10 for 10 minutes before being moved directly to the microscope. Cells were imaged in phenol red free media (FluoroBrite DMEM, 25 mM HEPES, 1% PenStrip) and a soluble fluorescent dye was added to the media (either Alexa Fluor647 or Alexa Fluor488 carboxylic acid). Experiments without rotavirus use FluoroBrite DMEM, 25 mM HEPES, 1% PenStrip with 5% FBS. *Virus labeling* – The triple layer particle (TLPs) was diluted to 0.4 mg/ml in a total volume of 50 μl using HNC (20 mM HEPES pH 8.0, 100 mM NaCl, 1 mM CaCl2), and 5.5 μl of 1 M NaHCO3 (pH 8.3) was added. This solution was mixed with 0.5 μl of 0.76 mg/ml Atto488 NHS ester. The reaction proceeded at room temperature for 1 hour before quenching it with 5 μl of 1 M Tris pH 8.0. The labeled TLPs were then buffer exchanged into a solution containing 20 mM Tris pH 8.0, 100 mM NaCl, and 1 mM CaCl2 using a Zeba Spin Desalting Column. *Recoated particle formation and labeling:* TLPs, DLPs, VP7, and VP4 were purified as previously described [47]. VP7 and VP4 were expressed in Sf9 cells infected with a baculovirus vector. VP7 was purified by successive affinity chromatography on concanavalin A and monoclonal antibody (mAb159), specific for the VP7 trimer (elution by EDTA). Purified VP7 was desalted into a solution containing 2 mM HEPES (pH 7.5), 10mM NaCl, and 0.1mM CaCl2 (0.1HNC). For VP4, harvested cells were lysed by freeze-thawing and clarified by centrifugation after the addition of a completely EDTA-free protease inhibitor (Roche). VP4 was precipitated by the addition of ammonium sulfate to 30% saturation, pelleted, and resuspended in a solution containing 20 mM Tris (pH 8.0) and 1mM EDTA, and then loaded onto a HiTrap Q column (GEHealthcare), and eluted in a gradient of 10 to 150 mM NaCl. Pooled fractions containing VP4 were dialyzed O.N on a 20 mM Tris (pH 8.0), 100 mM NaCl and 1 mM EDTA buffer.

VP7 and DLP were labeled as previously described[45]. VP7 was brought to 1.7 mg/ml in a total volume of 100 ul using 0.1HNC, and 11.1 ul of 1 M NaHCO3 (pH8.3) was added. This solution was mixed into 0.71 ul of 0.76 mg/ml Atto560-NHS ester. The reaction proceeded at room temperature for 1 h before quenching with 10 ul of 1 M Tris (pH 8.0). The labeled VP7 was then desalted into a solution containing 2mM Tris (pH 8.0), 10 mM NaCl, and 0.1 mM CaCl2 (0.1TNC). 50 ug DLP was brought to a volume of 100 ul in HN, to which 11.1 ul 1 M NaHCO3 (pH 8.3) was added. This solution was then added to 1.5 ul of 0.5 mg/ml of Atto647N-NHS ester. The reaction proceeded 1h and room temperature before quenching with 10 ul of 1 M Tris (pH 8.0). The sample was then desalted through a 0.5 ml Zeba Spin Desalting Column into a solution containing 20mM Tris (pH 8.0) and 100mM NaCl (TN). We distributed 45 μg of DLPs in HNE equally among five 1.5 ml conical tubes (0.5 μl per tube). We first added 1 M sodium acetate, pH 5.2 to a final concentration of 100 mM and then added 82 μl VP4 (stored at 1.8 mg/ml) to a final concentration of 0.9 mg/ml in the final reaction volume, resulting in a 33-fold excess of VP4 monomer over a total of 180 sites on DLPs. A 0.1 mg/ml aprotinin solution was added to the samples to a final concentration of 0.2 μg/ml followed by incubation at 37 °C for 1 h. Required amounts of VP7 (7.14 μl stored at 1.26 mg/ml in HNE) to achieve a 2.3-fold excess of VP7 monomer over a total of 780 sites on DLPs were premixed with 0.1 volumes of TC buffer (20 mM Tris, 10 mM $CaCl_2$, pH 8.0) and 0.1 volumes of 1 M sodium acetate pH 5.2 for 15 min before adding them to the DLP-VP4 mixture. Samples were incubated for 1 h at room temperature and then quenched by the addition of 0.1 volumes of 1 M Tris pH 8.0. Recoated particles from the five tubes were combined, and TNC was added to a final volume of 2.5 ml. rcTLPs were separated from excess VP4 and VP7 by ultracentrifugation at 4 °C in a Beckman Coulter rotor TLS 55 at 50,000 rpm for 1 h. We removed 2.0 ml of the supernatant, returned the volume to 2.5 ml with TNC, and pelleted again. Supernatant was carefully removed so that 100 to 200 μl remained. The rcTLP pellets were resuspended in the remaining buffer and stored at 4 °C.

**Insulin labeling for SDCM imaging.** Human insulin was labeled with Atto-655-NHS ester. Human insulin Atto-655-NHS (LysB29Atto-655-HI) ester was prepared following previous publications [61] [62]. In short, Human insulin (21 mg, 0.0036 mmol, 3 equivalents) dissolved in 0.1 M tris Buffer (0.2 mL) with pH adjusted to 10.5 for complete dissolution, Atto-655-NHS ester (1.0 mg, 0.00122 mmol, 1.0 equivalent) in DMF (0.3 mL) was added by drops over 5 minutes to the Human insulin (HI) solution, followed by stirring for 15 minutes. The reaction was monitored by LCMS. Subsequently, the reaction mixture was diluted by 2.0 mL of milliQ water and pH adjusted to 7.8. Product was isolated using RP-HPFC, using Biotage SNAP ultra-column (C18, 30 g, 25 um). CH3CN/H2O mixed with 0.1% formic acid was used as eluents at a linear gradient of 5-50 % CH3CN over 20 minutes at a flow rate of 25 mL/min. Each fraction was analyzed through LCMS. Monosubstituted products were collected separately, CH3CN was removed at reduced pressure using a rotary evaporator, followed by lyophilized (LysB29Atto-655-HI yield: 5.6 mg, 79 %).

**LLSM imaging and experimental setup.** Rotavirus, EEA1-mScarlett. NPC1-Halo-JFX646, and AP2 tracking experiments were carried out using an in-house built LLSM as in previously published work [5,7]. The LLSM imaging ran in sample scan mode with 0.25 µm spacing between each plane along the z-imaging axis producing molecular videos consisting of 3D volumes using a dithered multi-Bessel lattice light sheet illumination. The exposure times and frame rates are listed per experiment in the Supplementary table 1. The resulting z-stacks were deskewed, followed by detection and linking across frames of punctuate light point sources producing sets of xyzt-trajectories using an automated tracking algorithm implemented in MATLAB based on least-squares numerical fitting of a 3D Gaussian as previously described [2,7].

**SDCM imaging.** An inverted spinning disk confocal microscope (SDCM) (Olympus SpinSR10, Olympus, Tokyo, Japan) was used for all 2D imaging of insulin. The SDCM uses an oil immersion 60x objective (Olympus) and numerical aperture of 1.4 connected to a CMOS camera (photometrics PRIME 95B) with an effective pixel size of 183 nm x 183 nm. Prior to imaging ~10000-20000 HeLa cells were grown at 37 ◦C, 5% CO2 in Ibidi IbiTreat 8-well plates for two days. LysoTracker® Green DND-26 using commercial stock concentration diluted 1:20.000 and added to incubate for 1 hour (37 ◦C, 5% CO2) prior to imaging. Lastly, for imaging insulin, 0.05 mg/mL LysB29Atto-655-HI was added to the HeLa cells to incubate for 1 hour (37 ◦C, 5% CO2) prior to imaging. Before any SDCM imaging experiments, wells are washed 3 times with fresh, pre-heated 10 mM HEPES in the HBSS buffer. For simultaneous SPT of insulin and compartments dual imaging was performed using lasers of 640 nm and 488 nm. SDCM live-cell imaging was performed with 30,4 ms exposure and 3 EM gain in SDCM streaming settings, resulting in 36 ms between frames including lag-time. The insulin was recorded with laser power 100% with 640 nm laser, and compartments were recorded with a laser power of 10% for LysoTracker® Green DND-26 for a total of 2000 frames at 37 ◦C. For tracking of LysB29Atto-655-HI in-house tracking scripts[8] were used with object diameter of 9 pixels, search range of 5 pixels, gap closing of 1 frame with mean-multiplier manually evaluated between 0.6 and 1. Post-processing of tracks was done using thresholds of; ecc < 0.3, intensity > 0 and duration > 20, in addition a logistic regression model trained to differentiate detection made in videos with/without insulin using detection features directly from tracking script was used to further filter detections.

## Supplementary Files

This is a list of supplementary files associated with this preprint. Click to download.

- SIDeepSPT.docx
- SupVid1RotavirusescapeDeepSPTv3.mov
- SupVid2Rotavirusescapev5.mov
- SupVid3NPC13DmovieDeepSPT.mov
